# Rapidly Self-Renewing Human Multipotent Marrow Stromal Cells (hMSC) Express Sialyl Lewis X and Actively Adhere to Arterial Endothelium in a Chick Embryo Model System

**DOI:** 10.1371/journal.pone.0105411

**Published:** 2014-08-21

**Authors:** Harris E. McFerrin, Scott D. Olson, Miriam V. Gutschow, Julie A. Semon, Deborah E. Sullivan, Darwin J. Prockop

**Affiliations:** 1 Xavier University of Louisiana, Biology Department, New Orleans, Louisiana, United States of America; 2 Program in Regenerative Medicine, University of Texas Medical School at Houston, Houston, Texas, United States of America; 3 Stanford Department of Bioengineering, Stanford University, Stanford, California, United States of America; 4 Center for Stem Cell Research and Regenerative Medicine, Tulane University School of Medicine, New Orleans, Louisiana, United States of America; 5 Department of Microbiology and Immunology, Tulane University School of Medicine, New Orleans, Louisiana, United States of America; 6 Texas A & M Health Science Center College of Medicine Institute for Regenerative Medicine at Scott & White, Temple, Texas, United States of America; University of California, San Diego, United States of America

## Abstract

**Background:**

There have been conflicting observations regarding the receptors utilized by human multipotent mesenchymal bone marrow stromal cells (hMSC) to adhere to endothelial cells (EC). To address the discrepancies, we performed experiments with cells prepared with a standardized, low-density protocol preserving a sub-population of small cells that are rapidly self-renewing.

**Methods:**

Sialyl Lewis X (SLeX) and α4 integrin expression were determined by flow cytometry. Fucosyltransferase expression was determined by quantitative realtime RT-PCR. Cell adhesion assays were carried out with a panel of endothelial cells from arteries, veins and the microvasculature *in vitro*. *In vivo* experiments were performed to determine single cell interactions in the chick embryo chorioallantoic membrane (CAM). The CAM is a well-characterized respiratory organ allowing for time-lapse image acquisition of large numbers of cells treated with blocking antibodies against adhesion molecules expressed on hMSC.

**Results:**

hMSC expressed α4 integrin, SLeX and fucosyltransferase 4 and adhered to human EC from arteries, veins and the microvasculature under static conditions *in vitro*. *In vivo*, hMSC rolled on and adhered to arterioles in the chick embryo CAM, whereas control melanoma cells embolized. Inhibition of α4 integrin and/or SLeX with blocking antibodies reduced rolling and adhesion in arterioles and increased embolism of hMSC.

**Conclusions:**

The results demonstrated that rapidly self-renewing hMSC were retained in the CAM because they rolled on and adhered to respiratory arteriolar EC in an α4 integrin- and SLeX-dependent manner. It is therefore important to select cells based on their cell adhesion receptor profile as well as size depending on the intended target of the cell and the injection route.

## Introduction

Human multipotent mesenchymal stromal cells, also known as mesenchymal stem cells or hMSC, are a potentially promising therapeutic treatment for a variety of diseases [1]–[3]. The cells can differentiate into multiple cell types, but they frequently repair tissues and prevent tissue damage without much evidence of engraftment. Instead the repair is mediated by secretion of paracrine factors, immunomodulation and the induction of angiogenesis and arteriogenesis [4]–[9]. For many therapeutic purposes, intravenous (IV) and intraarterial (IA) injection are the most attractive routes of administration since the cells appear to have the capacity to home to injured organs [10]–[14].

Numerous studies have demonstrated that hMSCs accumulate in the lungs of mice following IV injection. There is still debate whether hMSC interact with the endothelium similarly to that of leukocytes or whether they simply become embolized in capillary beds downstream from the injection site. The interactions of leukocytes with EC is a multi-step process that involves rolling and adherence of the leukocytes followed by extravasation into surrounding tissues (for review, see [15], [16]). Ruster et al. [17] first demonstrated that hMSC rolled and then adhered to human umbilical vein endothelial cells (HUVEC) in a parallel plate flow chamber. Experiments with blocking antibodies indicated that the adherence of hMSC to HUVEC required both P-selectin and VCAM-1/VLA-4 (α4/β1 integrin) interactions. Using intravital microscopy of the mouse ear, they also demonstrated that hMSC rolled on and adhered to postcapillary venules in a P-selectin dependent manner; however, the hMSC they employed did not express P-selectin glycoprotein ligand (PSGL). More recently, two groups reported that hMSC did not express SLeX, a carbohydrate component of PSGL and ligand for other selectins, in sizeable amounts. They then demonstrated that engineering the cells to express SLeX increased the percentage of hMSC that rolled on activated venular endothelium *in vitro* and *in vivo*, decreased rolling velocity and increased adherence of the cells to endothelium [18], [19]. In 2013, Nystedt and colleagues investigated cell adhesion molecule involvement in lung retention of hMSC in a mouse model. The group reported that hMSC from bone marrow (BM-MSC) and from umbilical cord (UCB-MSC) grown at low passage and low confluency expressed several cell adhesion molecules. In their study, a significant percentage of UCB-MSCs and BM-MSCs expressed SLeX, Podocalyxin-like protein 1 (PODXL1), **α** 6 integrin, **α**4 integrin and fibronectin. UCB-MSCs expressed higher levels of alpha6 and alpha4 integrins and were cleared from the lung more rapidly than BM-MSCs. Further, treatment of BM-MSC and UCB-MSC with pronase to cleave extracellular protein epitopes resulted in decreased cell signal in the lung at 15 hours post-injection [20].

Methods to study interactions of hMSCs with endothelium of the lung are limited due to the location of the lung within the body and its movement [16]. The chick embryo is a unique model system with an easily accessible external respiratory organ, the chorioallantoic membrane (CAM). The CAM has been used extensively to determine how cancer cells interact with the vasculature *in vivo* [21]–[30]. The CAM and mouse lung are characterized by similar vessel size and complexity [31]. Both organs have a highly anastamosing system of capillaries which creates a relatively small pressure differential across the capillary bed in comparison to that of muscle. This smaller pressure differential may increase the retention and survival of cancer cells in both of these model systems [30] [32]. Additionally, the CAM receives a large portion of the blood flow [33], allowing for the observation of a larger number of injected cells in comparison to the mouse ear.

In the experiments reported here, we employed hMSC grown with a standardized protocol of low density culture that preserves a sub-population of the cells that are rapidly self-renewing [34], [35]. In contrast to preparations utilized in several earlier publications, the hMSC expressed SLeX and α4 integrin. We first determined the ability of the hMSC to adhere under static conditions *in vitro* to EC from arterial, venous and microvascular sources and found that hMSC preferentially adhered to unstimulated arterial EC from two sources compared to venular endothelium and microvascular endothelium from the dermis. We then examined adherence and rolling of hMSC *in vivo* in the chick embryo CAM because *in vivo* microscopy provides a unique perspective allowing for the observation of biological phenomena in a respiratory organ in real time under physiological conditions.

Our results indicated that hMSC had a marked tendency to adhere to and roll on arteriolar vessels in the CAM. Rolling and adherence to arteriolar endothelium was significantly reduced by treatment with fucoidin, a pan-selectin inhibitor, and by injection of blocking antibodies against SLeX and α4 integrin expressed on the hMSC.

## Materials and Methods

### Ethics Statement

All animal procedures were approved by the Institutional Animal Care and Use Committee (IACUC) at Tulane University and conformed to the requirements of the Animal Welfare Act. PBMC were obtained from the New Orleans Blood Center and hMSC were obtained from the Texas A&M Institute for Regenerative Medicine without identifiers and were therefore IRB exempt.

### Chemicals

Rhodamine Lens Culinaris Agglutinin and VectaShield with DAPI were obtained from Vector Laboratories (Burlingame, CA). Fluospheres, Quant-iT pico green, Cell Tracker green and Texas Red-conjugated bovine serum albumin (BSA) were obtained from Molecular Probes (Eugene, OR). Fucoidin was obtained from Sigma Chemical Company (St. Louis, MO).

### Preparation of Cells

Low passage number of human umbilical vein EC (HUVEC), human iliac artery EC (HIAEC), human pulmonary artery EC (HPAEC), human aorta EC (HAEC), human cardiac artery EC (HCAEC) and human microvascular EC from dermis (HMVEC-D) were obtained from Lonza, Inc. (Walkersville, MD) and cultured in either of two commercial media (EGM2 or EGM2-MV; Lonza). The melanoma cell line B16F1 was obtained from the ATCC (Rockville, MD) and cultured following the recommendations of the supplier.

Extensively characterized preparations of hMSC [35] were obtained from the Texas A&M Institute for Regenerative Medicine (http://medicine.tamhsc.edu/irm/msc-distribution.html) and met the requirements defining multipotent mesenchymal stromal cells [36]. Briefly, the cells were shown to be multipotent for differentiation through 3 passages, were negative for hematopoietic markers (CD34, CD36, CD117 and CD45), and were positive for CD29 (95%), CD44 (>93%), CD49c (99%), CD49f (>70%), CD59 (99%), CD90 (99%), CD105 (99%) and CD166 (99%). Frozen vials containing 10^6^ passage 1 hMSC were plated in 150 cm^2^ tissue culture plates for 24 hours to recover adherent viable cells. The cultures were washed with PBS and adherent cells were lifted with 0.25% trypsin and 1 mM EDTA at 37 °C for 3 minutes. The cells were replated at 100 cells/cm^2^, incubated for 6 to 7 days until approximately 70 to 80% confluent, and lifted with trypsin/EDTA. For further expansion, the cells were replated and incubated under the same conditions. The culture medium was complete culture medium: alpha-MEM (Gibco-BRL, Rockville, MD), 20% FBS (lot selected for rapid growth; Atlanta Biologicals, Norcross, Ga), 1% penicillin, 100 µg/mL streptomycin, and supplemented with 2 mM L-glutamine (Gibco).

### Static Adhesion Assay

For static adhesion assays, EC (passages 3 to 4) were grown to confluence on 6-well collagen coated plates (BD Bioscience). hMSC were lifted with 0.25% trypsin/1 mM EDTA and incubated with 1 µM CellTracker Green (Molecular Probes, Eugene, OR) according to manufacturer's recommendation. About 6×10^5^ hMSC were added per well to the confluent EC at 37°C. After 15 minutes, wells were gently washed three times with PBS, and adherent cells were counted in 10 fields (100-fold magnification) per well using a fluorescence camera (Zeiss) with a software program (Metamorph Software; Molecular Devices, Sunnyvale, CA).

### Flow Cytometry

Approximately 5×10^5^ hMSC were labeled by resuspending the cells in 500 µL PBS and incubating for 30 minutes at room temperature (RT) with 50 µL of IgM control antibody (Zymed Laboratories, San Fransisco, CA), 10 µL of anti-human integrin α4-phycoerythrin (clone 9F10; BD Bioscience, San Jose, Ca), 20 µL anti-human SLeX IgM (clone CHO131) [37], or 20 µL anti-human P-selectin IgG-FITC (clone 9E1, both from R&D Biosystems, Minneapolis, MN). Cells were then washed three times with 500 µL PBS and, where appropriate, incubated with 10 µL goat anti-mouse IgM AlexaFluor 488 (Invitrogen, Carlsbad, CA) for 30 minutes. After washing three times with PBS, cells were analyzed by flow cytometry (Cytomics FC 500; Beckman Coulter, Fullerton, CA) with CXP software.

### Real-time PCR

Total RNA was extracted from low passage, subconfluent cultures of hMSC from 3 individual donors and from peripheral blood mononuclear cells (PBMC) using the RNeasy Mini Kit (Qiagen; Valencia CA). A total of 500 ng of cellular RNA was used for cDNA synthesis using Bio-Rad iScript cDNA synthesis kit (Bio-Rad, Hercules, CA). PCR reactions were performed using TaqMan Universal PCR master mix (Life Technologies Applied Biosystems, Carlsbad, CA) and the iCycler Real-Time PCR detection system (Bio-Rad) with the following primer sets (Life Technologies Applied Biosystems): FUT4 (Assay ID Hs01106466_s1), FUT7 (Assay ID Hs00823637_g1), selectin P ligand (SELPLG) (Assay ID Hs04276253_m1). Negative controls, including cDNA reactions without reverse transcriptase or RNA and PCR mixtures lacking cDNA were included in each PCR. Samples amplifying at a C_T_ value greater than 35 were considered below the detectable range.

### Embryo Injections

Fertilized white leghorn chick embryos were obtained from Charles River Laboratories (Wilmington, MA) and stored at 4°C. To promote development, the embryos were incubated for 11 days at 37°C in a humidified incubator with rocking. The shell was opened over the air pocket and the skin overlying the CAM was rendered transparent by the addition of mineral oil. Prior to injection, hMSC were lifted with trypsin/EDTA, washed with PBS, counted, and re-suspended for 30 min in phenol red-free alpha MEM without FBS but containing 10 µM CellTracker Green for 1 hour or with 3 µL/mL PicoGreen (Molecular Probes). The cells were isolated by centrifugation, washed with and resuspended in phenol red-free alpha MEM. Where cells were treated with blocking antibodies, hMSC were incubated with 50 µg/mL anti-SLeX antibody or with 5 µg/mL anti-α4 antibody or with both antibodies (clones cho131 and 2b4, respectively; R&D) for 1 hour at 37 °C 5% CO_2_. About 5×10^5^ hMSC, 5×10^5^, 5×10^5^ B16F1 cells or 5×10^4^ beads were resuspended in 50 µL phenol red-free αMEM with 0.1% BSA containing 5 µL (5 mg/mL) Texas Red BSA (Molecular Probes) to provide vessel contrast. The egg was inverted over an inverted microscope stage, and the cell/bead suspension was injected into a large CAM vein, distinguished from an artery by the lighter color of the oxygenated blood. Ten 100 ms exposure images were captured every second for at least 3 minutes to capture velocity or every minute for 10 minutes to capture localization at 40× magnification with a charged-coupled device camera (ORCA-ER; Hamamatsu Photonics, Bridgewater, NJ) on an inverted microscope (Eclipse TE200; Nikon). Images were captured using Wasabi imaging software (Hamamatsu Photonics) and later exported for analysis in Metamorph software (Molecular Devices) or ImageJ software (National Institutes of Health, Bethesda, Maryland). Areas containing arteries and opposing veins separated by a capillary bed were chosen for analysis. Vessel diameter, vessel length, cell diameter, cell velocity (length of streak or point to point) and the number of cells per minute were measured over 10 minutes. Vmax was set as the speed of the fastest cell in each vessel. Cell rolling was defined by cells moving less than the critical velocity (V_crit_) = V_max_×(D_cell_/D_vessel_)/(2−(D_cell_/D_vessel_) [38], where D is the diameter of the cell or the vessel. Cells were counted as adherent or embolized if they remained stationary for longer than one frame. Cells that remained in vessels larger than their diameter were defined as adhered to arterioles or veins whereas cells located either in the ends of tapering arterioles or in the capillary bed were defined as embolized. Images from at least 5 chicks were measured per condition, and all images were scored in a blinded fashion. For 3-dimensional images, lens culinaris agglutinin lectin conjugated with rhodamine was injected 10 minutes after injection of cells. Z-stacked images were acquired on an upright spinning disk confocal microscope (IX70; Olympus) using StereoInvestigator software (MBF Bioscience, Williston, VT).

### Statistical Analyses

Data are expressed +/− SEM. ANOVAs with Tukey post-tests and two-way ANOVAs with Bonferri post-tests were performed to determine statistical significance (Prism 5 Software for Mac). Data on adhesion *in vitro* were from experiments repeated three times and data on observations *in vivo* were from experiments repeated at least four times.

## Results

### hMSC adhere to EC derived from human arteries, veins and microvasculature under static conditions *in vitro*

**Figure 1:**
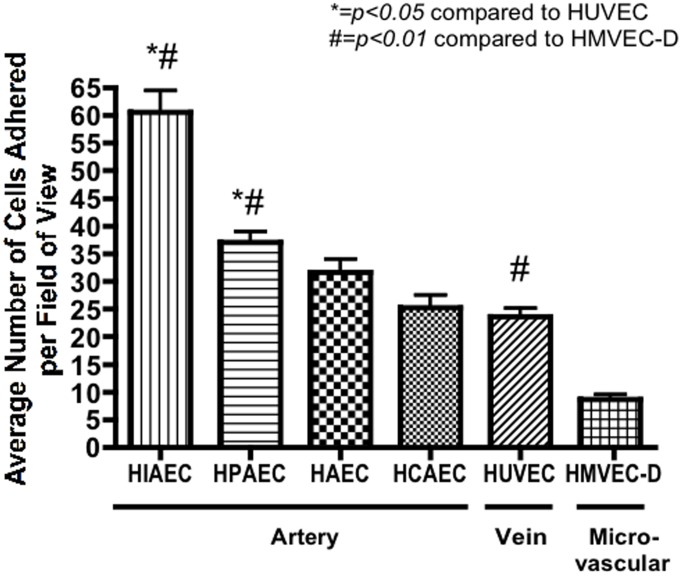
Low passage hMSC adhere to human arterial, venous and microvascular EC *in vitro.* Values are means +/− SEM of adhered hMSC per field (100× magnification; n = 10). Data were obtained from three experiments with hMSC from two preparations from two different donors of marrow adhered to commercially available, pooled EC from either venous, arterial or dermal microvascular origin.

Static adhesion assays are useful tools to understand interactions between cellular adhesion receptors and their ligands [39]. To determine whether hMSC selectively adhere to different EC, hMSC from two donors were added to confluent cultures of human EC from different sources (Figure 1): HIAEC, HPAEC, HAEC, HCAEC, HUVEC and HMVEC-D. After 15 minutes incubation at 37 °C, unbound cells were washed away, and the number of adherent cells per field in each well were counted using an inverted microscope (Figure 1). hMSC preferentially adhered to EC from the different vessels in the following order: iliac artery (60.6±4.0) > pulmonary artery (37.1±1.9) > aorta (31.6±2.4) > cardiac artery (25.2±2.4) ≥ umbilical vein (20.7±1.5) > dermal microvasculature (8.7±0.9). The values for binding to EC from iliac artery and pulmonary artery were significantly higher than the values for EC from umbilical vein (*p*<0.001 and <0.01 respectively) and microvasculature (*p*<0.001). The binding to EC from umbilical vein was also significantly greater than to dermal microvasculature (*p*<0.01). To determine whether the results varied with different preparations of hMSC, the assays were repeated using preparations obtained from three different donors of bone marrow. There were no significant differences in binding by different preparations of hMSC to EC from human iliac artery (data not shown). These results indicated that the hMSC adhered to all cells tested and bound preferentially to EC from two arterial sources compared to EC from dermal microvasculature and to EC from human umbilical cord.

### Experimental design to determine hMSC-vascular interactions in the chick embryo CAM

**Figure 2:**
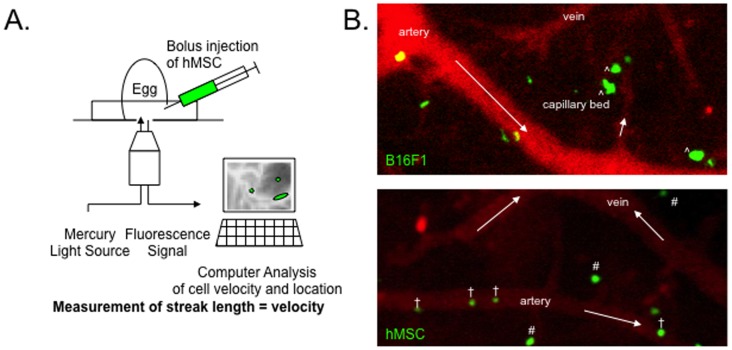
Real time assay of cells in vessels of the chick embryo CAM. **A.** Schematic for injecting cells or beads into a large vein of the CAM and capturing images for 3 to 10 minutes at either 40× or 100× magnification. **B. (upper panel).** Green B16F1 melanoma cells were primarily embolized in the capillary bed and had distorted morphology (∧). **(lower panel).** Green hMSC retained a regular morphology and were found both within arteries (†) and within the capillary beds (#). Images taken 10 minutes after injection of the cells. Arrows indicate direction of blood flow. Magnification 100×.

To expand the *in vitro* observations *in vivo*, we used the chick embryo CAM as described by Chambers et al. and MacDonald et al. [28], [32], [40], [41] (Figure 2A). In this system, arteries were defined in time-lapse digital images as vessels in which darker blood flowed (arrows) from larger vessels to smaller vessels and veins as vessels in which lighter blood flowed from smaller vessels to larger vessels (Figure 2B). We infused either hMSC or controls of B16F1 melanoma cells that were fluorescently labeled into the CAM vasculature and obtained digital images 10 minutes later. The results confirmed previous reports that the melanoma cells rapidly embolized in the tapering arterioles and capillaries of the CAM (Figure 2B. upper panel). In the process, the cells were distorted in shape and underwent noticeable clasmatosis or membrane blebbing. The melanoma cells did not adhere to larger vessels. In contrast, hMSC were found primarily in arteries and arterioles with diameters larger than the diameters of the cells (Figure 2B, lower panel). The differences were not explained by differences in cell size: the melanoma cells were 20.5 µm±0.7 and the hMSC 19.65 µm±0.6. Also in contrast to the melanoma cells, the hMSC retained their shape without the release of vesicles that were detectable at 100× magnification after the injection of B16F1 cells.

### hMSC adhere to vessels in the chick CAM whereas melanoma cells embolize

**Figure 3:**
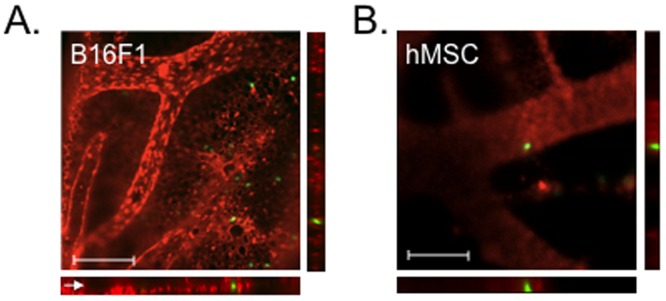
3-Dimensional images of cells in rhodamine-labeled vesicles of chick embryo CAM. Orthologous projections of z-stacked photomicrographs of the CAM at 200× magnification. Crosshairs indicate cell of interest. **A.** B16F1 melanoma cells primarily embolized in the overlying capillary plexus (arrowhead) and at the ends of tapering arterioles. **B**. An hMSC, retaining its shape, adhered in a large vessel (dashed lines) lying beneath the capillary plexus.

To confirm that hMSC adhere within large vessels *in vivo*, we captured images through the z-plane of the CAM and employed deconvolution and 3-dimension rendering to improve visualization of cell location with respect to the vasculature. We first injected labeled melanoma cells or hMSC into a CAM vein, and 10 minutes later injected lens culinaris agglutinin lectin conjugated with rhodamine to improve visualization of vessels (Figure 3). After 10 additional minutes, the CAM was excised and stored at 4°C until analyzed. Orthogonal projections of z-stacked images indicated that hMSC were often found within larger vessels beneath the capillary layer (Figure 3B), indicating that hMSC actively adhered to endothelium of respiratory blood vessels *in vivo*. In contrast, the melanoma cells were localized to the overlying capillary plexus (Figure 3A). At this timepoint, there was no evidence that the hMSC invaded the endothelial layer of the vessels. (Z-stacks used to produce the orthogonal projects are presented as Videos S1 for B16F1 and S2 for hMSC).

#### hMSC are cleared from the circulation more slowly than melanoma cells or 10 µm beads

**Figure 4:**
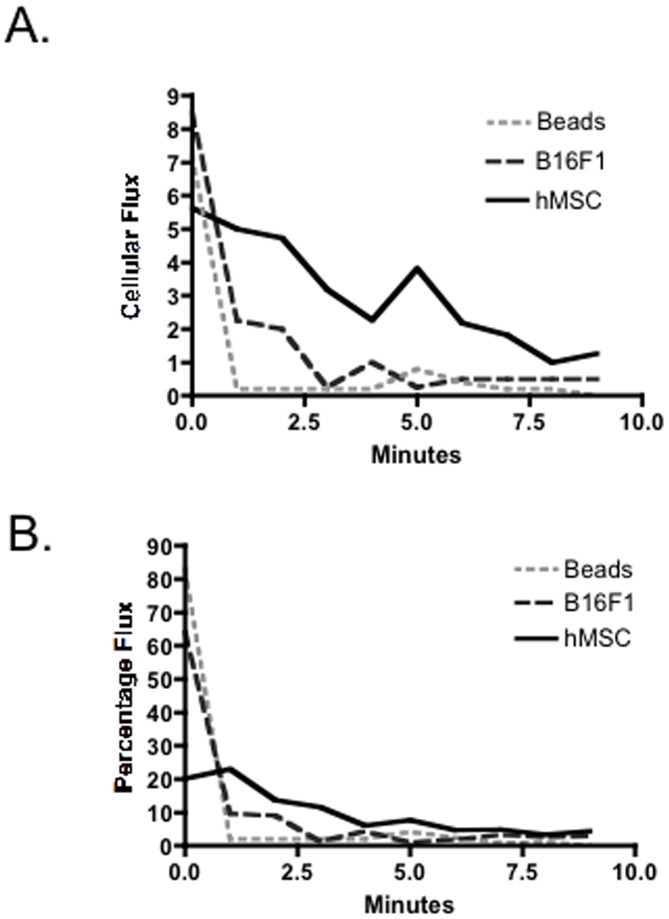
Clearance from the circulation of hMSC, melanoma cells and 10 µm inert beads. Inflexible inert 10 µM beads and B16F1 are cleared from circulation faster than hMSC. **A.** Values for cellular flux calculated as the average number of cells or 10 µm beads counted within vessels each minute in the CAM at 100× magnification. B. Values expressed as percentage flux were calculated as cells or beads in one minute as % of total observed in 10 minutes (n≥6).

Gross inspection of time-lapse photomicrographs suggested that hMSC persisted in the circulation longer than melanoma cells. To examine clearance of hMSC from the circulation of the chick embryo, we injected hMSC and then followed their appearance in the vessels of the CAM every minute for 10 minutes. For comparison we injected melanoma cells or inert beads that were 10 µm in diameter under the same conditions. Figure 4A represents total cellular flux and figure 4B represents flux as a percentage of the total number of cells or beads observed. In the first minute after the injection, a smaller percentage of the hMSC per field were observed (20.2% +/− 4.6; n = 7) than B16F1 melanoma cells (64.1 +/− 14.3, p<0.001) or beads (83.0 +/− 9.6, p<0.001). By both measurements, 10 µm beads were cleared from circulation within the first minute following bolus injection. B16F1 cells were cleared with similar kinetics. In contrast, hMSC cellular and percentage flux per minute was initially lower than B16F1 cells and beads and remained higher than beads and B16F1 throughout the remaining observation period. These data suggested that some of the hMSC infused into veins in the CAM had circulated through the heart and been slowed in their progress due to interactions with blood vessels proximal to the CAM. When expressed either as cellular or percentage flux, the data over the 10 minute interval demonstrated that the hMSC were cleared from the circulation more slowly than B16F1.

### Low passage, rapidly dividing hMSC express SLeX and α4 integrin

**Figure 5:**
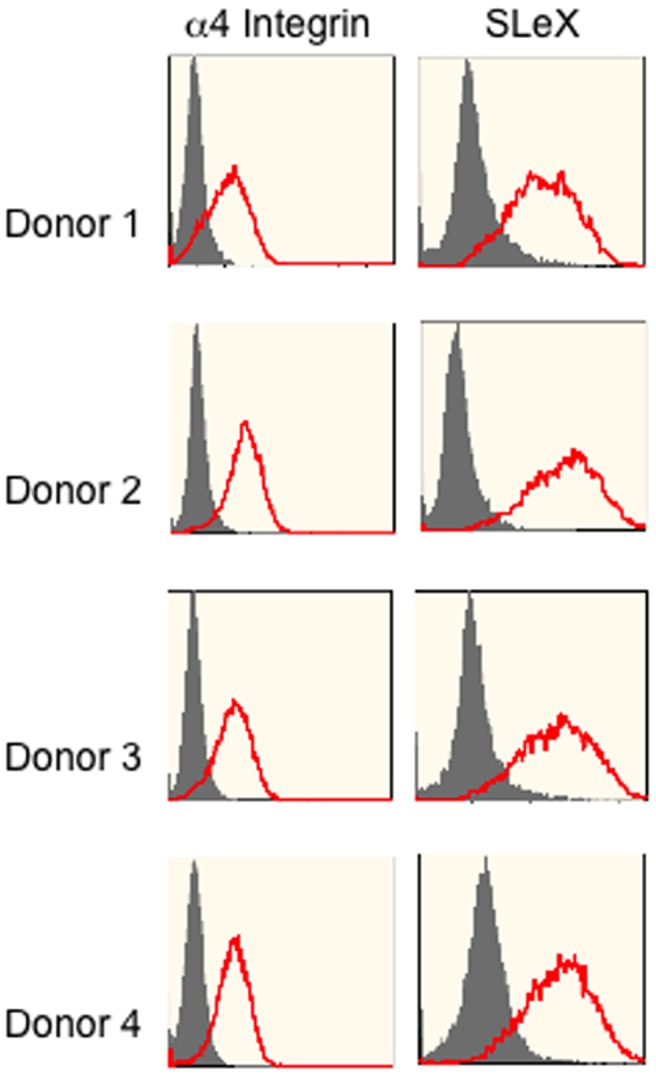
Low passage hMSC express α4 integrin and SLeX. hMSC derived from four preparations from four different donors were assayed for expression of α4 integrin and SLeX by flow cytometry. Passage 1 cells were plated overnight to recover adherent viable cells and then re-plated at 100 cells/cm^2^. The cells were harvested when 70 to 80% confluent.

Because hMSC had been previously shown to interact with postcapillary venules in a P-selectin and VLA-4 (α4/β1 integrin)-dependent manner [17], we analyzed low passage hMSC from four donors to determine whether they expressed adhesion molecules involved in this pathway. hMSC from all four donors expressed both SLeX and α4 integrin at passage 2 (Figure 5). The expression of both was moderately intense and expressed by nearly 100% of cells in cultures that were harvested at day 6 to 7 and 70 to 80% confluency. qRT-PCR was performed to determine whether FUT 4 and 7, enzymes that form SLeX, were expressed in samples generated from 3 donors. FUT 4 was expressed in samples from all 3 donors tested with average C_T_ values of 30.8, 30.5 and 31.3 compared with an average C_T_ value of 28.2 from peripheral blood mononuclear cells. PSGL and FUT 7 were not expressed.

### Low passage hMSC roll on arterial EC *in vivo* through SLeX and α4 integrin interactions

To define the molecules involved in the rolling and adhesion of hMSC to vessels *in vivo*, we first examined the ability of fucoidin, a pan-selectin inhibitor, to inhibit binding. After intravascular injection of 5 mg/kg fucoidin 5 minutes prior to injection of hMSC into the vascular system, hMSC did not appear to adhere or roll on arteries compared with sham-injected embryos. Instead they moved faster through the vasculature, and lodged primarily in the capillary plexus (data not shown).

**Figure 6:**
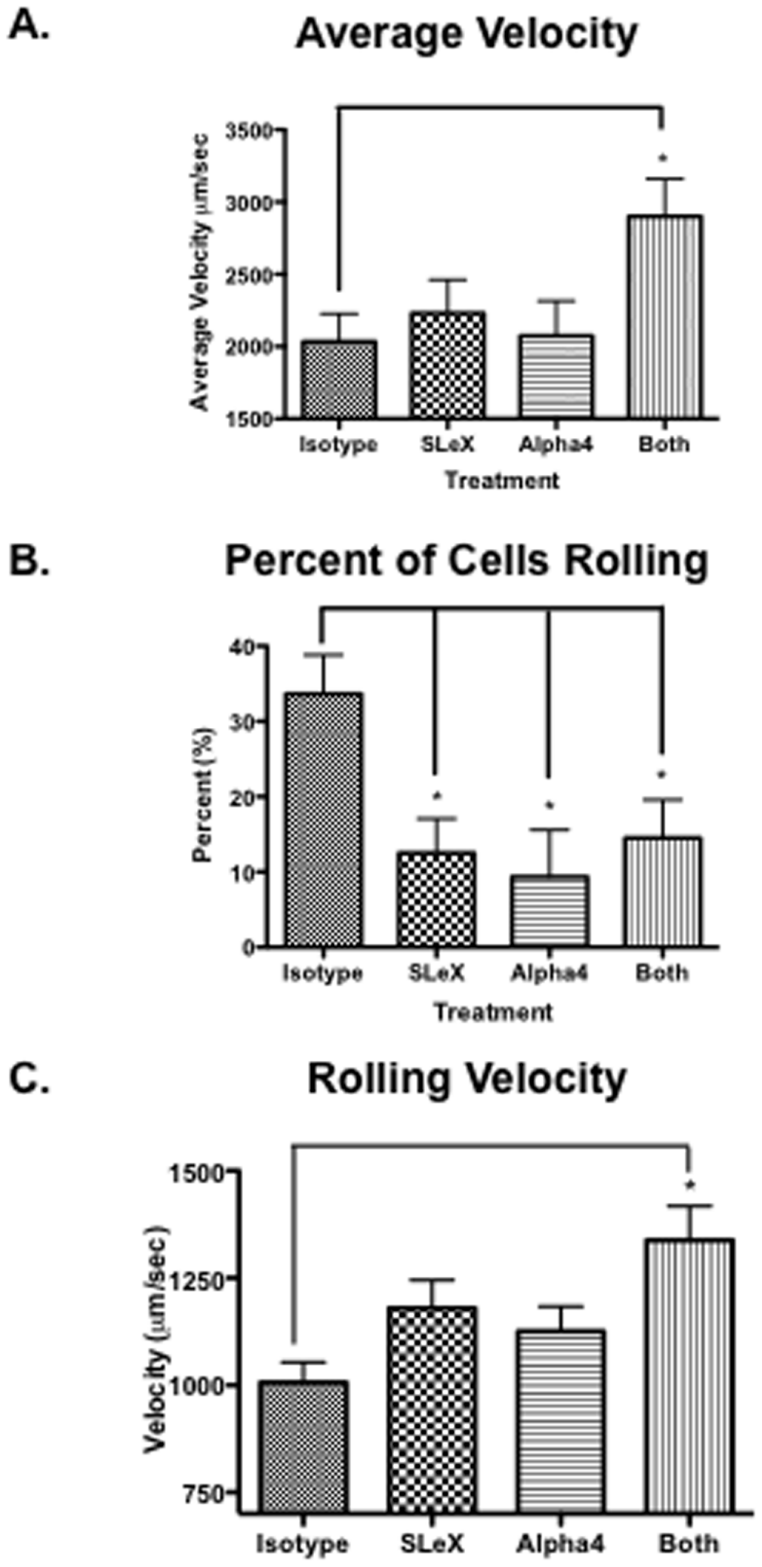
Effects of anti-SLeX and anti-α4 integrin on MSC rolling on arterial endothelium *in vivo*. **A.** Per cent of hMSC rolling without or with pre-treatment with anti-SLeX, anti-VL α4 integrin, or both antibodies. * = *p*<0.05 **B.** Average velocity of the cells. V_max_ did not significantly change among treatment conditions (average = 3682.4±29.1 µm/sec). Measuring fluorescence signal, D_hMSC_ was 20.3±0.4 µm in these experiments. D_artery_ was 93.5±4.1 µm and was not significantly different among treatment groups. * = *p*<0.05. **C**. Rolling velocities of the cells. * p<0.01.

To determine which adhesion molecules were involved, we then tested blocking antibodies against previously identified candidate adhesion molecules. After treatment of the hMSC with anti-SLeX, anti-α4 integrin or both antibodies together, fewer cells were seen to adhere or roll following bolus injection suggesting that SLeX and α4 integrin contributed to hMSC-vascular interactions. The results were not explained by the antibodies increasing the size of the cells, since assays of the fluorescence signal indicated that the average diameter of hMSC was 20.3±0.4 µm in these experiments, and the average arterial diameter was 93.5±4.1 µm and was not significantly different among treatment groups. To confirm the observations, cellular velocity was measured following treatment with blocking antibodies (Figure 6A). Average cell velocity increased when cells were treated with anti-α4 integrin or SLeX antibodies, and treatment with both antibodies significantly increased average velocity above that seen with each antibody independently, suggesting a synergistic effect (isotype 2034.1±192.7 µm/sec, α4 integrin 2074.2±241.9 µm/sec, SLeX 2230±231.1 µm/sec, both 2901.2±258.7 µm/sec; *p<0.05*). V_max_ did not vary significantly among treatment conditions (Isotype 3945.1±473.0 µm/sec, α4 integrin 3114.8±384.1 µm/sec, SLeX 3298.2±80.2 µm/sec, both 4371.8±385.7 µm/sec) suggesting that blood flow characteristics were not altered between each treatment condition. When measured as a percentage of total observed cells, 34%±5 of cells treated with isotype antibodies rolled in arteries (Figure 6B). By comparison, 9%±6 of cells treated with the α4 integrin antibody, 13%±5 of cells treated with SLeX and 15%±5 of cells treated with both antibodies rolled (*p*<0.05 for all values compared with isotype antibody-treated cells). Similar results were observed when the mean number of rolling cells was determined in arteries per millimeter of vessel. 6.78±2.5 cells rolled per millimeter of artery when treated with isotype antibody compared with 1.4±0.63 for SLeX antibody-treated cells, 2.69±1.92 for α4 antibody-treated cells and 1.75±0.55 for cells treated with both antibodies. Rolling velocity was significantly higher for hMSC treated with both antibodies compared with to hMSC treated with isotype antibody (Figure 6C). Based upon these data, we concluded that low passage hMSC rolled in arteries of the chick embryo CAM and that this rolling was synergistically dependent upon interactions involving SLeX and α4 integrin

#### Low passage hMSC adherence in arteries is synergistically dependent on SLeX and α4 integrin and is not donor-dependent

**Figure 7:**
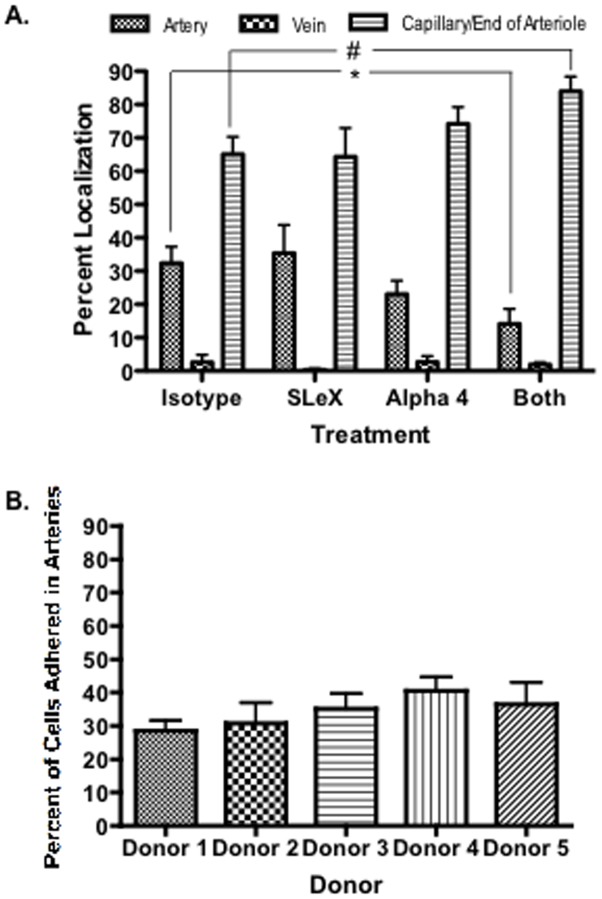
Distribution of hMSC to arteries/arterioles, veins and capillaries/end arterioles in the CAM. **A.** Distribution of hMSC compared to lymphocytes and effects of pre-treatment with anti-SLeX and/or anti-α4 integrin (n = 5). **B**. Distribution in arteries of hMSC from 5 preparations from 5 different donors of marrow repeated 5 times.

To define further the roles of SLeX and α4 integrin *in vivo*, we counted the number of fluorescently-labeled cells localized within the arteries, veins and capillary bed of the CAM three minutes after bolus injection. Cells that were clearly localized within macrovessels larger than the diameter of the cell were assigned to arteries or veins. Cells localized either in the tapering ends of arterioles or within the capillary plexus were counted as embolized. (Figure 7A) 32.3%±5.0 of cells treated with isotype antibody adhered within arteries. Treatment with both antibodies significantly decreased arterial localization to 14.1%±4.6 while the percentage of hMSC embolizing in the capillaries or in the ends of tapering arterioles increased from 65.1%±5.2 to 84.0%±4.4. Treatment with anti-SLeX or anti-α4 antibody alone did not significantly decrease arterial localization (35.4%±8.5, 23.1%±4.0) or increase embolism (64.3%±8.7, 74.2%±5.0). When the mean number of adherent cells was determined per mm^2^ of the field of view for arteries, veins and embolism at the ends of arterioles and in capillaries, the number of embolized cells increased significantly from 4.68±0.69 to 12.08±3.60. Venular localization was not significantly altered by treatment with blocking antibodies when measured as either a percentage of total cells or as the mean number of cells per mm^2^. To determine whether binding to arterial endothelium was donor or preparation dependent, we repeated the experiment with 5 donors and with human lymphocytes (Figure 7B). Roughly equivalent numbers of hMSC adhered to arterial endothelium. Thus, we concluded that the hMSC adhered to arterial EC by synergistic binding through SLeX and α4 integrin. Of note, when embryos were injected with confluent, high passage hMSC from the same donors as used in the figures above, the cells formed large aggregates and embolized in the the vessels (not shown).

## Discussion

The static, *in vitro* assays presented in this manuscript demonstrated that hMSC exhibit baseline adhesiveness to EC from arteries, veins and microvasculature. In this assay hMSC preferentially adhered to unstimulated EC from arteries compared to EC from umbilical vein or microvasculature. The results were extended by assays *in vivo* with time-lapse microscopy of the chick embryo CAM. Experimental results obtained in the chick embryo have been shown to be predictive of results obtained in mammalian models [42], [43]. Consistent with a previous report by MacDonald et al, we observed that following injection into the CAM of chick embryos, B16F1 melanoma cells failed to adhere and embolized in small vessels [32]. In contrast to melanoma cells, we observed a much larger fraction of hMSC in arterioles with diameters larger than the hMSC after injection under the same conditions. Up to 30% of low passage hMSC were found in arterioles after intravenous injection of the cells where they were shown to roll along the endothelium in an SLeX- and α4 integrin-dependent manner. These results are consistent with previous reports demonstrating that hMSC associate with arteries under normal conditions [44]–[46]. In the studies presented in this manuscript, rolling of hMSC in chick embryo venules was not observed; however, this may be due to the experimental design. Since the critical rolling velocity in a vessel was determined by the fastest moving cell in that vessel, if only one or two cells passed through a particular venule, we were unable to determine whether the cells were rolling. Adherence, however, could be determined by the localization of single cells. The inability of a large percentage of injected cells to pass through the capillary plexus has been observed in other animal models [55] and may be due to embolism and/or adherence to the arterial or capillary vasculature.

We have previously published that sub-confluent hMSC express moderate levels of α4 and β1 integrins and that α4 integrin was the only integrin to vary significantly based on donor preparation [47], [48]. Subtle variations in the properties of different preparations of hMSC may have important implications for therapeutic uses of the cells. For example, the tendency of hMSC to form lethal pulmonary emboli in mice was shown to vary with different protocols for preparing the cells that altered the expression of anti-cell adhesion surface proteins such as podocalyxin-like protein [49].

The chick embryo CAM can readily be visualized and has been used extensively to determine how cancer cells interact with the vasculature *in vivo* [21]–[30]. The CAM receives a large portion of the total blood flow in the egg [33], allowing for the observation of a larger fraction of injected cells than either the mouse ear or mesentery. Circulating cells in chick blood express selectin ligands including SLeX carbohydrate moieties and integrins, and chick embryo endothelium expresses appropriate binding partners for these molecules including selectins, VCAM-1 and fibronectin necessary for homing[27], [50]–[54].

Similar to the experimental results of Ruster et al., we found that genetically and biochemically unmodified hMSC were capable of rolling on and adhering to endothelium [17]. The results presented here differ however in that Ruster et al. focused on venous EC (HUVEC) and post-capillary venules. Our results also differ from the results of several studies in which hMSC were found not to express SLeX and that fewer than 50% hMSC expressed α4-integrin [17]–[19], [56]. These differences may be explained by their use of high passage (up to passage 20) cultures instead of the early passage (2–3) and low density cultures used here that are enriched for smaller and rapidly-replicating hMSC [34], [35]. In support of this assertion, Greenberg et al. demonstrated that the most naive CD34+ hematopoetic stem cells rolled avidly in a PSGL-dependent manner in a flow chamber on immobilized P-selectin and that their ability to roll and adhere decreased as the cells differentiated [57]. Additionally, we observed that when hMSC obtained from confluent, high passage cultures were injected into the chick embryo, the cells aggregated and formed emboli that occluded larger vessels (unpublished data). Similarly to Nystedt and Kerkela, we found that hMSC from bone marrow utilize α4 integrin for adhesion to respiratory endothelium. In the CAM model system, SLeX and α4 integrin were both involved in hMSC rolling and adherence whereas the contribution played by SLeX was not apparent in the mouse lung [20], [58]. Additional experiments will be necessary to determine whether SLeX and α4 integrin are the primary adhesion molecules utilized by hMSC in various disease states and in different organs.

Many observations support the association of MSC with arteries [44]. The results presented in this manuscript are in agreement with studies in mice showing that murine MSC home to perivascular sites and contribute to arteriogenesis and decrease arterial resistance [6]. Murine MSC deficient in apolipoprotein E (ApoE) incorporate into the aorta and reduce angiotensin II-induced aortic aneurysm formation in ApoE-deficient mice [59]. We have also observed that hMSC and to a greater degree, serum-deprived hMSC are highly angiogenic and incorporate into the vasculature of the chick embryo CAM [9].

Although lung adherence by MSCs is generally considered a negative consequence of IV injection, several major diseases involve inflammation of the lung including interstitial lung disease, asthma, emphysema, chronic obstructive pulmonary disorder, cystic fibrosis and acute respiratory distress syndrome. In these cases, increased concentration of the cells following IV injection would presumably be beneficial due to their immunosuppressive characteristics and thus warrants further study; however, PODXL1, α6 integrin and other cell adhesion molecules should be examined both individually and in combination.

## Supporting Information

Video S1 **Videos were compiled from z stack images at 200× magnification displaying B16F1 cells and hMSC in large vessels and in the overlying capillary plexus.** Vessels were counterstained after cell injection by lens culinaris agglutinin rhodamine injection. Images were acquired on a spinning disk confocal microscope using StereoInvestigator software. (S1) B16F1 cells were found primarily in the overlying capillary plexus. (S2) hMSC located beneath the capillary plexus in a larger vessel.
(MP4)

Video S2 **Videos were compiled from z stack images at 200× magnification displaying B16F1 cells and hMSC in large vessels and in the overlying capillary plexus.** Vessels were counterstained after cell injection by lens culinaris agglutinin rhodamine injection. Images were acquired on a spinning disk confocal microscope using StereoInvestigator software. (S1) B16F1 cells were found primarily in the overlying capillary plexus. (S2) hMSC located beneath the capillary plexus in a larger vessel.
(MP4)

Video S3 **Video was compiled from time-lapse images taken every second at 100× magnification.** Green hMSC treated with isotype control antibody can be seen free-flowing or rolling in an artery contrasted with Texas Red BSA.
(MP4)
